# Cost-effectiveness analysis of pulse oximetry screening for critical congenital heart defects following homebirth and early discharge

**DOI:** 10.1007/s00431-018-3268-x

**Published:** 2018-10-17

**Authors:** Ilona C. Narayen, Arjan B. te Pas, Nico A. Blom, M. Elske van den Akker-van Marle

**Affiliations:** 1 Department of Pediatrics, Division of Neonatology, Leiden University Medical Center, Leiden, the Netherlands; 2 Department of Pediatrics, Division of Pediatric Cardiology, Leiden University Medical Center, Leiden, the Netherlands; 3 Department of Biomedical Data Sciences, Section Medical Decision Making, Leiden University Medical Center, Leiden, the Netherlands

**Keywords:** Cost-effectiveness, Congenital heart defects, Screening, Neonatology, Pulse oximetry, Health economy

## Abstract

Pulse oximetry (PO) screening is used to screen newborns for critical congenital heart defects (CCHD). Analyses performed in hospital settings suggest that PO screening is cost-effective. We assessed the costs and cost-effectiveness of PO screening in the Dutch perinatal care setting, with home births and early postnatal discharge, compared to a situation without PO screening. Data from a prospective accuracy study with 23,959 infants in the Netherlands were combined with a time and motion study and supplemented data. Costs and effects of the situations with and without PO screening were compared for a cohort of 100,000 newborns. Mean screening time per newborn was 4.9 min per measurement and 3.8 min for informing parents. The additional costs of screening were in total €14.71 per screened newborn (€11.00 personnel, €3.71 equipment costs). Total additional costs of screening and referral were €1,670,000 per 100,000 infants. This resulted in an incremental cost-effectiveness ratio of €139,000 per additional newborn with CCHD detected with PO, when compared to a situation without PO screening. A willingness-to-pay threshold of €20,000 per gained QALY for screening in the Netherlands makes the screening likely to be cost-effective.

*Conclusion*: PO screening in the Dutch care setting is likely to be cost-effective.

**Table:** 

**What is Known:** • *Pulse oximetry is increasingly implemented as a screening tool for critical congenital heart defects in newborns.* • *Previous studies suggest that the screening in cost-effective and in the USA a reduction in infant mortality from critical congenital heart defects was demonstrated.*	
**What is New:** • *This is the first cost-effectiveness analysis for pulse oximetry screening in a setting with screening after home births, with screening at two moments.* • *Costs of pulse oximetry screening in a setting with hospital and homebirth deliveries were €14.71 and is likely to be cost-effective accordint to Dutch standards.*

## Introduction

Pulse oximetry (PO) screening to detect critical congenital heart defects (CCHD) in newborns has been studied widely in the past years and was proven to be accurate, safe, easy, and acceptable in settings with delivery and screening in hospital [6, 12, 15, 23, 30]. Cost-effectiveness analyses performed in studies from the USA and UK also suggest that the screening might be cost-effective in their setting [21, 25].

Congenital heart defects are the most common congenital defect, affecting approximately 8 per 1000 live births. One quarter of all congenital heart defects are critical and require surgery or catheter intervention in the first month of life [9]. Timely diagnosis of these CCHD, before signs of cardiovascular collapse, is pivotal in reducing morbidity and mortality. With prenatal screening, around 50–80% of CCHD can be detected [24, 32]. Postnatal physical examination of remaining cases is hampered by the absence of clinical signs in the first days of life [3, 13, 19]. PO can be added to the regular screening program (prenatal ultrasound and postnatal examination) in order to reduce the cases with late diagnoses. It is known that a timely diagnosis of CCHD improves the chances of a favorable outcome with less mortality and morbidity [3].

Although cost-effectiveness studies were performed in the USA and UK in settings with screening in hospital, costs might be different in settings with different perinatal care systems [21, 25]. For example, the Netherlands is unique with a high rate of home births (18%) and discharge within 5 h after an uncomplicated vaginal delivery in hospital [27, 29]. Screening in this setting requires performance of PO at home by community midwives, as well as a referral system for positive screenings. Recently, an accuracy study in the Dutch perinatal care was performed including 23,959 infants [16]. We aimed to estimate the additional costs of PO screening in the Dutch perinatal care system, taking into account personnel time and equipment. The costs and cost-effectiveness of a situation with PO screening were compared to the current setting, with effectiveness measured in terms of timely diagnosis (before death or signs of acute cardiovascular collapse).

## Materials and methods

### Screening strategies

The situation with PO screening as an adjunct to clinical examination was compared to usual care in which no PO screening was performed.

In the situation with PO screening, PO was added to physical examination of newborns and performed at home or in hospital at two time periods: at least 1 h after birth and on day 2 or 3 of the infant’s life. Infants with abnormal screenings were referred to the pediatrician for physical examination and a cardiac ultrasound was made in case of persistent abnormal oxygen saturations in the absence of a non-cardiac explanation.

**Fig. 1:**
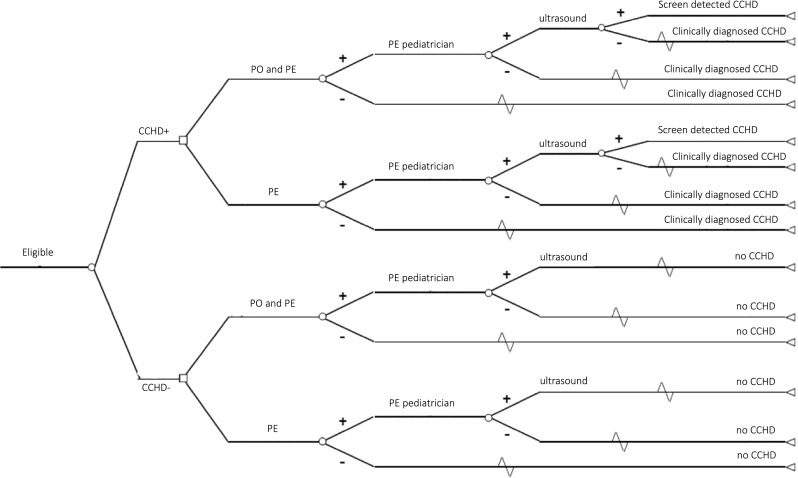
Schematic representation of screening pathways. CCHD critical congenital heart defect, PE physical examination, PO pulse oximetry

In a situation without PO screening, a physical examination is performed by the midwife or the obstetric nurse. If this examination has an abnormal result, referral to the pediatrician for examination including a cardiac ultrasound will take place. In Fig. 1, both screening strategies are shown.

### Clinical data

Clinical data for the situation with PO screening were obtained from the Pulse Oximetry Leiden Amsterdam Region (POLAR) study. The protocol and results of this study are published in another article [16]. The study included 23,959 infants, 6 infants with CCHD were detected, 5 by abnormal PO results, and 1 due to clinical symptoms, while 5 CCHD were missed (sensitivity 54.5%, specificity 99%). The false-positive rate was 0.9%, but 61% of these infants had significant other pathology. Also, the percentage of referred neonates transported by an ambulance in a situation with PO screening were obtained from the POLAR study.

For the situation without PO, the number of physical examinations by midwives and obstetric nurses was assumed to be the same as in the situation with PO screening. Data on referrals were obtained from a review of patients’ records before the introduction of PO. From all infants with CCHD that were not detected during antenatal anomaly scan, the records were reviewed in order to assess when the infants became symptomatic, if there was a timely diagnosis, and if postnatal physical examination revealed symptoms.

The percentage of infants without CCHD with a false-positive result in a situation with physical examination alone, was assumed to be 0.4%, based on data derived from previous studies where the false-positive rate of physical examination in health newborns varied between 0.3 and 0.5% [6, 7, 33]. The clinical parameters used in the model are summarized in Table 1.

**Table 1:** Model parameters for a situation with and without PO screening added to physical examination

	Situation with PO screening		Situation without PO screening	
Parameter	Value	Source	Value	Source
CCHD positive children				
% screen positive by clinical examination and/or pulse oximetry	54.5%	POLAR	25.8%	Chart review
% transported by ambulance if screen positive	50.0%	POLAR	50.0%	POLAR*
% physical examination if screen positive	100%	POLAR	100%	Expert opinion
% cardiac ultrasound if screen positive	100%	POLAR	100%	Expert opinion
CCHD negative children				
% screen positive by pulse oximetry	0.9%	POLAR	–	–
% screen positive by clinical examination	0.4%	Assumption based on previous studies literature (3, 33, 34)	0.4%	Assumption based on previous studies literature (3, 33, 34)
% transported by ambulance if screen positive	2.2%	POLAR	2.2%	POLAR*
% physical examination if screen positive	100%	POLAR	100%	Expert opinion
% cardiac ultrasound if PO screen positive	18.1%	POLAR	–	–
% cardiac ultrasound if PE screen positive	100%	Expert opinion	100%	Expert opinion

*PE* physical examination

*Assumed to be the same as in PO/PE screening group

### Costs of screening and referral

The cost evaluation is performed from a healthcare perspective. All reported costs were converted to values for 2017, by means of the consumer price index [28, 34]. As the cost of physical examination was assumed to be the same in the situation with and without PO, only the additional costs of PO were assessed.

A total of 28 community midwives recorded the time of 190 PO screenings. Also, the duration of the parent information talks during the antenatal visit and at the first screening moment was measured. We assumed that these time measurements were also representative for PO screenings performed by obstetric nurses. Personnel costs of the screening were obtained by multiplying the time duration of the screenings by the hourly gross salary costs of respectively midwives (€59, personal communication Royal Dutch Organization of Midwives (KNOV)) and obstetric nurses (€32) [34].

Cost of equipment was based on the purchase price of the new pulse oximeter devices and reusable sensor with wraps requested at the vendor (PM10N handheld pulse oximeters with reusable OxiMax sensors, Medtronic, Ireland, Dublin). We assumed a depreciation period of 8 years for the pulse oximeter and 6 months for the sensors. Cost of annual maintenance were assumed to be 5% of the purchase price [34]. The mean number of devices in midwife practices and hospitals was obtained from participating practices and hospitals in the study [16]. This was multiplied by the number of midwife practices and hospitals in the Netherlands and divided by the total number of infants screened per year to obtain the costs of the device per infant screened [4, 18, 20].

The percentage of neonates with a repeat PO screening was obtained from the POLAR study. Respectively 1.0 and 0.3% tests at the first and second moment of screening were repeated.

Referral costs included the cost of an outpatient visit to the pediatrician (€102), ambulance transport (€621), and costs of cardiac ultrasounds (€ 490) for the subgroup of neonates with persistent abnormal oxygen saturations without a non-cardiac explanation [17, 34].

### Analysis

In the base case analysis, costs and effects of both the situation with and without PO screening are compared using the model parameters described above for a cohort of 100,000 neonates with a gestational age ≥ 35 weeks, that were not monitored with pre- and post-ductal SpO_2_ in the first 24 h of life and in whom no cardiac ultrasound was performed. The cost-effectiveness ratio was obtained by dividing the difference in costs in a situation with and without PO screening by the difference in number of timely diagnosed infants with CCHD. We identified the costs per cases identified, which is a partial economic evaluation according to the Drummond et al. classification [5]. Although not the preferred cost-utility analysis, our analysis may represent important intermediate stages in the understanding of the cost and consequences of PO screening [5].

Additionally, sensitivity analyses were performed to assess the impact of alternative assumptions for the model parameters on the incremental cost-effectiveness ratio.

In these sensitivity analyses, the cost and effects of performing one measurement in the first hours after birth instead of two measurements was assessed. Performing only one measurement leads to a lower sensitivity of 45.5%, a lower percentage of children without CCHD receiving a positive PO result (0.8%) and lower costs of screening. Furthermore, the effects and costs were assessed if a sensitivity of 70% was assumed for PO screening, which may also be likely for the Dutch situation [16, 32].

Also (univariate) sensitivity analyses on cost parameters were performed. In the base case analysis, a depreciation period of 8 years for the pulse oximeter was assumed, this was changed in a 5-year period in the sensitivity analysis, leading to higher material costs of screening (€4.32 per infant). The Dutch tariff for cardiac ultrasound in newborns is quite high compared to the costs assumed for the UK and the USA [21, 25]; therefore, also a sensitivity analysis with lower costs for cardiac ultrasound of €250 was performed.

Analyses were performed using Microsoft Excel (Microsoft, Seattle, WA) 2010 software.

## Results

### Screening costs

A total of 190 PO screenings were timed by community midwives. The mean screening time was 4.9 min (SD 2.7 min, range 1.0–15 min). The mean parental information time of 3.8 min (SD 2.5 min, range 1.5–12 min). The two screening moments and parental information together amount to time costs of €11.00 per infant screened. Costs of pulse oximeter devices and the reusable sensor with wraps amount to €3.71 per infant, resulting in additional costs of PO screening of €14.71.

### Effects and cost of screening with and without PO

In the situation without PO, 11 per 100,000 infants with CCHD were timely diagnosed. Adding PO resulted in an additional number of 12 CCHD per 100,000 infants. In the situation with PO screening, the estimated cost with the addition of PO screening and referral amounts to € 1,923,000 per 100,00 infants, of which the additional costs of PO screening account for €1,471,000 (Table 2), and the costs of referral were €452,000. In the situation without PO screening, costs of referral including ambulance transport, pediatrician visit, and cardiac ultrasound were € 252,000 per 100,000 infants. Therefore, the additional cost of screening and referral in a situation with PO screening were €1,671,000 per 100,000 infants (€1,923,000 minus €252,000) compared to a situation without PO screening (Table 2). The resulting incremental cost-effectiveness ratio, representing the additional cost per additional timely detected infant with CCHD, was € 139,000 (Table 3).

**Table 2:** Cost of PO screening and referral in a situation with and without the addition of PO to PE screening, per 100,000 infants (2017 €)

Cost category	Situation with PO screening	Situation without PO screening
PO screening	1,471,000	0
Referral	452,000	252,000
- Ambulance transport	25,000	9000
- Pediatrician	138,000	42,000
- Cardiac ultrasound	289,000	201,000
Total cost of screening and referral	1,923,000	252,000

**Table 3:** Cost and effects in a situation with and without the addition of PO to PE screening for different assumptions of the model parameters, per 100,000 infants (2017 €)

Sensitivity analysis	Situation with PO screening		Situation without PO screening		Cost-effectiveness ratio Costs per additional timely detected infant with CCHD
	Costs	Effects	Costs	Effects	
Only PO measurement at day 1	1,299,000	19	252,000	11	128,000
Higher sensitivity PO (70%)	1,677,000	30	252,000	11	86,000
Shorter depreciation period pulse oximeter (5 years)	2,025,000	23	252,000	11	148,000
Lower costs cardiac ultrasound (€250)	1,627,000	23	252,000	11	136,000
Base case	1,922,000	23	252,000	11	139,000

### Sensitivity analysis

The sensitivity analyses in which base case values of the model parameters were changed did not lead to important changes in the cost-effectiveness ratio, except for assuming a higher PO sensitivity, which resulted in a considerably lower cost-effectiveness ratio with a reduction of €53,000 per additional timely detected infant with CCHD (Table 3). Other factors of the sensitivity analysis, such as using only one PO measurement moment, led to a maximum difference €11,000 per additional timely detected infant with CCHD.

## Discussion

The additional costs of PO screening are €14.71 per screened newborn. Total additional costs of screening and referral are €1,671,000 per 100,000 infants. This would implicate that the annual costs for implementing PO screening in the Netherlands would be €2.4 million. With an estimate of 12 extra timely detected CCHDs per 100,000, this resulted in a cost-effectiveness ratio of €139,000 per timely diagnosis CCHD, when compared to the current management with antenatal anomaly scan and postnatal physical examination. A willingness-to-pay (WTP) threshold of €20,000 per gained QALY in the Netherlands for prevention indicates that PO screening in the Dutch care setting would be cost-effective if considerable savings in lifetime treatment and/or substantial gains in QALYs would be obtained per infant timely diagnosed with PO screening [31]. It is known that the improved techniques of pediatric cardiac surgery and catheter interventions have considerably improved the outcome of children with CCHD in the last decades, with an improved life expectancy and quality of life [10, 11]. However, exact and recent data on gained QALYs by timely diagnosis are lacking. The majority of infants with CCHD survive at least up to adulthood, and it is expected that the majority of them have normal life expectancy [11]. Recent data have also shown that the short-term morbidity, mortality and length of hospital stay are reduced in case of timely diagnosis of CCHD [3]. An analysis of the importance of timely diagnosis of CCHD, performed in the USA and based on a birth defect registry, stated that potentially preventable death occurred in 1.8% of infants with late detected CCHD, and that a late diagnosis was associated with more and longer hospital admissions, and higher inpatient costs [22]. Abouk et al. reported a reduction in early infant deaths from critical congenital heart disease from 33.4%, with an absolute decline of 3.9%, after states implemented mandatory screening compared with prior periods and states without screening policies [1]. Taking into account this reduction in mortality, Grosse et al. estimate the cost per life-year gained at $12,000 [8]. However, this estimate does not include long-term costs of care and education of survivors, nor costs for support and monitoring of screening programs. If 3.9 deaths per 100,000 births can be prevented, and assuming a life expectancy of 60 years with a utility of 0.75 (conservative assumption, lower than general population), a total of 164 QALYs can be gained per 100,000 births (83 QALYs with 3% discounting) [1, 10]. Combining this with the incremental costs for PO screening of €1,671,000, this results in a cost-effectiveness-ratio of €10,183 per QALY (or €20,000 with 3% discounting). This is likely to be acceptable according to Dutch Willingness-To-Pay threshold of €20,000 per gained QALY for prevention [31].

PO screening performed in hospital setting in the US costed $14.19 (2011) per screened newborn, which was less than the costs for metabolic (heel prick) screening and hearing screening in their setting [21]. In a cost-effectiveness analysis of PO screening performed in the UK, additional costs of PO screening were £6.24 (2009). In our screening protocol, part of the screenings were performed at home, with referral to hospital in case of a positive screening. Furthermore, we adopted a two-step screening strategy with PO measurements at two time points, causing higher personnel costs. These factors partly explain the higher costs of PO screening per newborn in our setting. Also, costs of referral, especially of cardiac ultrasounds, were assumed to be higher for the Dutch situation, which together with the higher screening cost attribute to the less favorable cost-effectiveness ratio compared to the UK estimate of £24,000 per extra timely diagnosis of CCHD when compared to physical examination alone. As shown in the sensitivity analyses, the prenatal detection rate of CCHD has a large impact on the cost-effectiveness ratio; a high prenatal detection rate of CCHD in our implementation study resulted in less CCHD detected postnatally, when compared to the other studies [16]. This increases the costs per additional detected case as well. The prenatal detection in our study might have been an overestimation of the actual prenatal detection rate, since it reflects the detection rate in a restricted area in the Netherlands in a timeframe of 1.5 years. No recent data were published on the prenatal detection rate of CCHD in the Netherlands, but a study performed in the same study area demonstrated a prenatal detection rate of 50% for CCHD in the period from 2002 to 2012 [32].

A strength of this cost-effectiveness analysis is that it was based on data acquired by a large primary accuracy study, with an additional time and motion study to assess time duration of screening and informing parents [16]. Although there was no concurrent control group with physical examination only, we were able to evaluate the accuracy by assessing a retrospective cohort from our own patient population from the period before PO screening was introduced. Although we did assess the additional costs per detected newborn with CCHD, we could not assess the long-term costs and benefits per QALY, which is of high importance for policy makers. No other cost-effectiveness analysis in other countries could assess this, however, due to lacking up-to-date long-term outcomes of children with CCHD. However, we made a calculation based on the recently published reduction in infant cardiac death following US state-wide implementation, which make the screening in our setting likely to be cost-effective with less than €20,000 per gained QALY. Another limitation is that we did not include treatment costs in this analysis, but studies have shown that the duration and amounts of hospital admissions is higher in case of late detection of CCHD [3, 22].

An extra value of PO screening is the detection of other pathology, such as infections and respiratory morbidity [16, 26]. Although these secondary targets were not included in cost-effectiveness analyses, it is likely that timely detection of these potentially life-threatening pathologies can reduce morbidity and mortality in neonates [2, 14].

## Conclusion

This cost-effectiveness analysis assessed PO screening in the Dutch perinatal care setting with a high rate of home births and early postnatal discharge. We calculated that PO screening in the Dutch care setting is likely to be cost-effective when considering the results of studies performed in the USA with a significant reduction in mortality following state-wide implementation. However, additional studies on life expectancy, quality of life, and treatment costs of children with CCHD are needed in the Dutch setting for exact calculations. The data we provided can be used by policy makers when considering implementation of PO screening.

## Abbreviations

CCHD: Critical congenital heart defects
PE: Physical examination
PO: Pulse oximetry
POLAR: Pulse oximetry Leiden-Amsterdam region
QALY: Quality adjusted life year
WTP: Willingness to pay
